# The importance of parental knowledge in the association between ADHD symptomatology and related domains of impairment

**DOI:** 10.1007/s00787-020-01579-4

**Published:** 2020-07-22

**Authors:** Tycho J. Dekkers, Hilde M. Huizenga, Jente Bult, Arne Popma, Bianca E. Boyer

**Affiliations:** 1 Department of Psychology, University of Amsterdam, Nieuwe Achtergracht 129B, 1018WS Amsterdam, The Netherlands; 2 Department of Forensic Psychiatry and Complex Behavioral Disorders, De Bascule, Academic Center for Child- and Adolescent Psychiatry, Duivendrecht, The Netherlands; 3 Department of Child and Adolescent Psychiatry, University Medical Center Groningen, University of Groningen, Groningen, The Netherlands; 4 Department of Child and Adolescent Psychiatry, Free University Medical Center (VUmc), Amsterdam UMC, Amsterdam, The Netherlands; 5 Amsterdam Brain and Cognition Center, University of Amsterdam, Amsterdam, The Netherlands; 6 Research Priority Area Yield, University of Amsterdam, Amsterdam, The Netherlands; 7 Veenlanden College, Mijdrecht, The Netherlands; 8 Psychologenpraktijk Kuin, Haarlem, The Netherlands

**Keywords:** Attention-Deficit/Hyperactivity Disorder (ADHD), Risk-taking behavior, Parental knowledge, Homework problems, Replication, Pre-registration

## Abstract

Parents of children with ADHD experience several difficulties while raising their children and report lower levels of knowledge about their children’s life and behaviors. A recent study found that low levels of parental knowledge mediated the association between ADHD symptoms and risk-taking behavior (RTB) in adolescents. The current study aimed to investigate this previous finding further by replicating it, by taking peer influence into account as additional social factor of importance and by extending it and also investigate the role of parental knowledge in the association between ADHD symptoms and homework problems. Three studies were performed: study 1 (*N*=234) replicated previous work on parental knowledge mediating the association between ADHD symptoms and RTB, study 2 (pre-registered, *N*=313) added peer influence, and study 3 (pre-registered, *N*=315) assessed whether parental knowledge mediated the association between ADHD symptoms and homework behavior. Parental knowledge consistently mediated the association between ADHD symptoms on one hand and RTB and homework problems on the other, and also predicted stronger resistance to peer influence. Because parental knowledge was repeatedly linked to ADHD-related problems, it seems promising to include parental knowledge in treatment of ADHD-related problems in adolescents, by improving the parent-child relationship. Future studies should test more directly how improvement of the parent-child relationship can be used to optimize parental knowledge, which in its turn reduces ADHD-related problems.

**Electronic supplementary material:**

The online version of this article (10.1007/s00787-020-01579-4) contains supplementary material, which is available to authorized users.

## General Introduction

Attention-Deficit/Hyperactivity Disorder (ADHD) is a neurodevelopmental disorder characterized by inattention, hyperactivity and impulsivity [1], occurring in 5.3-7.2% of the adolescents [2–4]. ADHD is associated with impairment in many domains of daily life [5, 6]. For example, ADHD is linked to a wide range of risk-taking behaviors, such as substance abuse, risky driving, gambling problems and sexual risk taking (see [7] for a review). Also, children and adolescents with ADHD encounter more academic problems and have poor learning outcomes relative to their peers without ADHD [8, 9]. ADHD symptoms and related problems do not only affect the child itself, but also challenge parenting. A meta-analysis demonstrated that parents of children with ADHD reported more parenting stress than parents of children without ADHD, and parental stress levels were associated with the severity of the ADHD symptoms of their children [10]. Also, relationships between children with ADHD and their parents are characterized by increased negativity [11, 12]. Altogether, this implies that parenting practices are related to ADHD symptoms and associated impairment.

An important aspect of parenting is parental knowledge, defined as “knowing where, how and with whom children spend their time” [13]. A longitudinal study revealed that ADHD symptoms measured at 8.5 years old predicted lower levels of parental knowledge 10 years later [14]. Low levels of parental knowledge can have severe negative long-term consequences like delinquency and substance use [13, 15]. Moreover, a recent study demonstrated that parental knowledge as reported by adolescents mediated the link between ADHD symptoms and general levels of risk-taking behavior (RTB) in adolescents: higher levels of ADHD symptoms were related to lower levels of parental knowledge, which were related to higher engagement in RTB [16]. In this Pollak et al. study, other parenting indices like disengaged relationships or behavioral autonomy did not mediate the association between ADHD symptoms and RTB.

The mediating role of parental knowledge in the association between ADHD symptoms and RTB observed by Pollak et al. is highly relevant to treatment – as it suggests to focus treatment of adolescents with ADHD on the increase of parental knowledge. However, the study by Pollak and colleagues is the first to demonstrate this mediation effect, had a relatively small sample size for assessing mediation (*N*=92) and focused only on the relation between ADHD symptoms, parental knowledge and RTB. Here, we report *three studies* that replicate the pioneering study by Pollak and colleagues and extend it by adding peer influence as additional mediator in the link between ADHD symptoms and RTB, and by investigating the possibility that parental knowledge also mediates the link between ADHD symptoms and other domains of impairment, in this case homework problems. The *first study* was a conceptual replication study, testing the mediating^1^ role of parental knowledge in the association between ADHD symptoms and RTB in a larger sample of adolescents (*N*=234) than the original study.

Parents may not be the only social factor influencing RTB in adolescents. As children mature into adolescents their need for autonomy grows and the influence of parents declines, whereas at the same time peer influence increases [17]. A wealth of experimental studies demonstrate that adolescents take more risks when they are either observed or explicitly encouraged to take risks by peers relative to when they are alone (e.g., [18–20]). Adolescents with ADHD may be particularly susceptible to peer influences as they experience social problems like peer rejection and involvement in deviant peer groups more often than adolescents without ADHD [21–24]. Therefore, in the *second study*, we again investigated the same mediation model (parental knowledge as potential mediator in the link between ADHD symptoms and RTB), now extending the design by investigating (low) resistance to peer influence as an alternative social factor that may result in RTB (i.e., resistance to peer influence was added as additional mediator). This time we used a pre-registered design and methodology, implying that the design and data-analytic approach were determined beforehand, and could not be changed during the course of the study. Pre-registration of studies prevents flexibility in data-analysis, and confirmation of previous findings using pre-registered methods increases confidence in the findings [25].

The influence of parental knowledge likely goes beyond RTB and potentially extends to other ADHD-related problems that typically occur during adolescence. Another domain in which parental knowledge may be relevant to understand ADHD-related impairment is homework problems. Adolescents with ADHD experience increased rates of academic problems [26] and relative to typically developing adolescents, they are more disorganized, lose homework more quickly, deliver more incomplete work and make more careless mistakes because of rushing through their homework [27, 28]. Homework problems are highly impairing for adolescents with ADHD [29], academic problems often persist with age [30] and the economic burden of academic problems related to ADHD is substantial [31]. Parents often have a supportive role in adolescents with homework problems [32–34] and parental knowledge is critical to support an adolescent in doing homework [35]. Paradoxically, adolescents high in ADHD symptoms likely need parental support in doing homework most, but parental knowledge is usually low in this group of adolescents [14]. In the *third study,* we therefore investigated if increased homework problems in adolescents high on ADHD symptoms may be mediated by parental knowledge as well.

In all three studies large samples were recruited from the general population. This is in line with several accounts regarding ADHD symptomatology as dimensional rather than categorical (cf. [16]; see [36] for a review), as well as with recent developments that encourage studying psychopathology in general as dimensional (e.g., Research Domain Criteria (RDoC); [37, 38]). An additional advantage of recruiting adolescents from the general population is that larger sample sizes can be obtained which are necessary to study the hypothesized mediation effects [39]. However, to ensure the inclusion of participants high on ADHD symptoms we recruited many of the participants at a so-called “multimedia college”. The creative nature of the school curriculum potentially attracts more adolescents high on ADHD symptoms [40], thereby purposefully oversampling the high end of the ADHD symptomatology continuum.

## Study 1

In the first study, we conceptually replicated the study by Pollak et al. (2017) on the mediating effect of parental knowledge on the association between ADHD symptoms and RTB. Following recent replication guidelines [41], the sample size of the current study was 2.5 times as large as that of the original study. Based on the original study we expected that (I) ADHD symptoms correlated positively with RTB; (II) ADHD symptoms correlated negatively with parental knowledge; (III) parental knowledge correlated negatively with RTB and (IV) there was an mediating effect of parental knowledge on the association between ADHD symptoms and RTB.

### Methods

#### Participants

Participants were 234 late adolescents (*see* Table 1), recruited at their school. Inclusion was based on age: adolescents between 16 and 19 years old could participate. There were no further in- or exclusion criteria. Adolescents were enrolled in the highest (32.9%) or second-highest (67.1%) level of high school in the Netherlands (i.e., pre-university or senior general secondary education), and therefore intelligence of all participants was likely to be above average. Most participating adolescents were from high socio-economic status neighborhoods, their ethnicity was predominantly Dutch (85% Dutch, 12% non-Western, 3% western; based on parents’ country of birth). The study was approved by the IRB of the University of Amsterdam. All participants gave written informed consent.

**Table 1:** Sample characteristics and descriptive statistics on outcome measures of study 1, 2 and 3. Range represents the minimum and maximum score in the sample, M = Mean, SD = Standard Deviation

	Study 1 (*N*=234)	Study 2 (*N*=313)	Study 3 (*N*=315)
Age, M (SD)	17.12 (.69)	17.23 (1.31)	17.11 (1.13)
Sex, % girls	53%	65%	61%
ADHD Rating Scale, M (SD; range)	17.23 (7.20; 3–40.5)	15.17 (8.78; 0–45)	15.40 (9.43; 0–50.5)
Parental Knowledge, M (SD; range)	26.21 (6.17; 2–36)	26.23 (7.71; 0–36)	25.34 (8.28; 0–36)
Risk-taking behavior, M (SD; range)	24.56 (12.64; 2–66)	18.89 (12.60; 0–73)	NA
Resistance to Peer Influence, M (SD; range)	NA	31.56 (4.30; 19–40)	NA
Homework Problems, M (SD; range)	NA	NA	16.60 (10.45; 0–56)

#### Materials

##### ADHD Self-report Scale

ADHD symptomatology was measured with the Dutch ADHD self-report scale for adults [42]. The scale consists of 23 items using a 4-point Likert scale, indicating the frequency of reported symptoms over the last six months. An example of an item is “I get distracted quickly”. Items resemble DSM-5 ADHD symptoms. Each symptom was operationalized in one item, except for five symptoms which were operationalized in two items. For the analyses, these double items were averaged (cf. [43]). Hence, scores potentially range from 0 to 54, with higher scores reflecting more ADHD symptoms. Internal and external validity were established [44]. In the current study, although the sample consisted of late adolescents instead of the adult population the scale was designed for, internal consistency was good, *α* = .85.

##### Risk Taking Questionnaire

A self-reported risk-taking questionnaire was administered, which consisted of 28 items describing different forms of risk-taking behavior (*see* Supplementary Materials 1 for all items). The questionnaire was based on the risk behavior questionnaire [45], the adolescent version of the DOSPERT [46] and the ADorTI (Pollak & Aran, unpublished data). An example of an item is ”How often do you use soft drugs?”. On each item, participants indicated how often they engaged in this behavior on a 5-point Likert scale, ranging from never to every week. Scores potentially range from 0 to 112, with higher scores reflecting more RTB. Internal consistency in the current study was good, *α* = .84.

##### Parental Knowledge Scale

To measure parental knowledge, nine items recommended by Stattin and Kerr [13] were used. On a 5-point Likert scale, adolescents described their parents’ knowledge of their life across multiple domains. Scores potentially range from 0 to 36, with higher scores reflecting more parental knowledge. An example of an item is “Do your parents know who you have as friends in your free time?”. Both internal consistency and test-retest reliability of this scale were good [13]. In the current study, internal consistency was adequate, *α* = .78.

#### Procedure

One author (JB) visited the participating school. Adolescents were briefed about the study in class; those willing to participate gave written informed consent and filled in a booklet containing demographic questions and all questionnaires. After the study, three gift cards were raffled among the participants as reward.

#### Data-analysis

With a power of .80, and coefficients similar to Pollak et al. (2017), a sample size of 71 would be sufficient [39]. However, as it is recommended for replication studies to obtain a 2.5 times larger sample than the original study [41], 230 participants were required (Pollak et al., 2017; *N*=92). Cronbach’s *α* was calculated to investigate internal consistency and normality was checked using a Kolmogorov-Smirnov test [47]. Correlation analyses were performed on the sum scores of the outcome measures. The mediation model with direct and indirect (via parental knowledge) effects of ADHD symptoms on RTB was tested with the SPSS PROCESS macro (Model 4 [48]; default 5000 samples bootstrapping; standardized values).

### Results

#### Correlation analyses

As ADHD symptoms, parental knowledge and RTB were not distributed normally (Kolmogorov-Smirnov *p*’s < .01), Spearman’s correlation analyses were performed. As expected, ADHD symptoms correlated positively with RTB, and negatively with parental knowledge, and parental knowledge correlated negatively with RTB (*see* Table 2).

**Table 2:** Spearman’s correlations between ADHD Symptoms, Parental Knowledge and Risk-Taking Behavior. All variables reflect sum scores. Note: ** *p* < .01, *** *p* < .001

	ADHD symptoms	Parental knowledge	Risk-taking behavior
ADHD Symptoms	−		
Parental Knowledge	−.19**	−	
Risk-Taking Behavior	.41***	−.34***	-

#### Mediation analysis

The mediation model was tested using the SPSS Process Macro model #4 [48], with ADHD symptoms as independent variable, parental knowledge as mediator, and RTB as dependent variable. All variables were standardized.

**Fig. 1:**
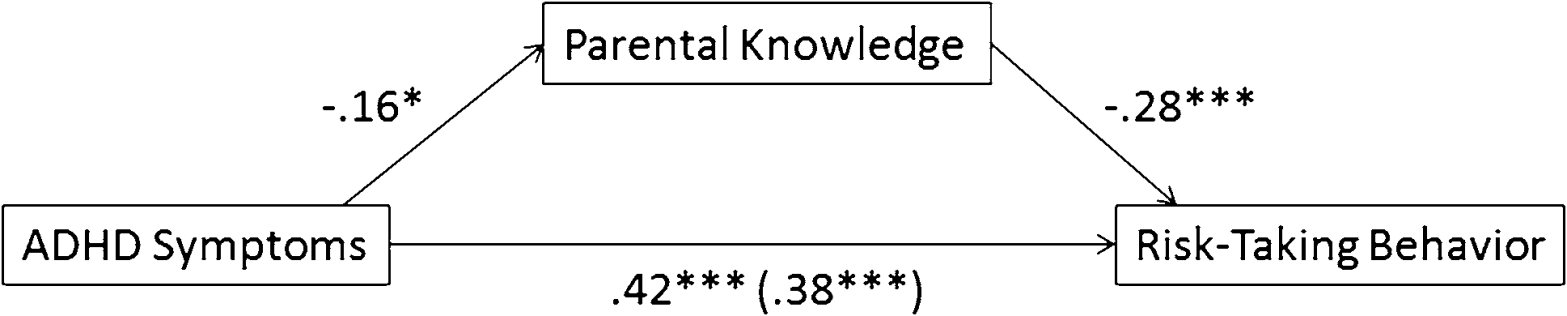
Mediation model: standardized regression coefficients for the relationship between ADHD Symptoms and Risk-Taking Behavior as mediated by Parental Knowledge. The direct effect of ADHD symptoms on Risk-Taking Behavior, controlling for Parental Knowledge, is in parentheses; * *p* < .05, *** *p* < .001

ADHD symptoms significantly predicted parental knowledge, *b* = -.16, *t*(232) = -2.42, *p* = .02; adolescents with more ADHD symptoms reported less parental knowledge (*see* Fig. 1). In a model that also included ADHD symptoms, parental knowledge significantly predicted RTB, *b* = -.28, *t*(231) = -4.88, *p* < .001; adolescents reporting more parental knowledge reported less RTB. The total effect of ADHD symptoms predicting RTB was significant, *b* = .42, *t*(232) = 7.04, *p* < .001; adolescents with more ADHD symptoms reported more RTB as well. Also after taking into account the mediating role of parental knowledge, ADHD symptoms still predicted RTB, *b* = .38, *t*(231) = 6.52, *p* < .001. The indirect effect was significant, as the bootstrap derived 95% confidence interval did not contain zero (.013, .095). The indirect effect explained 10.5% of the total effect, as established by dividing the standardized *b* of the indirect effect by the standardized *b* of the total effect [49]. The overall model including ADHD symptoms, parental knowledge and RTB was significant, *F*(2,231) = 39.11, *p* < .001, *R*^*2*^ = .25.

Regression analyses were also performed using bootstrapping of 5000 samples, as the data distribution was not normal: all *b*- and *p*-values were highly similar. Furthermore, the influence of outliers was minimal. After removing four outliers that deviated at least three standard deviations from the mean on one or more of the variables, the results changed only marginally. All correlations, and all pathways in the mediation model were still significant. Taken together, the hypothesis was supported, as there was an indirect effect of ADHD symptoms on RTB through parental knowledge.

### Discussion

The current study was a conceptual replication of the study by Pollak and colleagues (2017), which demonstrated that there is an indirect effect of parental knowledge on adolescents’ activities on the link between ADHD symptoms and risk-taking behavior. The current study, performed by an independent research group, in a different country, using a slightly different age range and using a 2.5 times larger sample size than the original study (cf. guidelines [41]), replicated these findings, which strengthens the robustness and generalizability of the original findings.

## Study 2

The aim of the second, pre-registered study was twofold: (a) to replicate the previous findings using a pre-registered design and (b) to investigate whether, apart from parental knowledge, (low levels of) resistance to peer influence also mediated the association between ADHD symptoms and RTB.

**Fig. 2:**
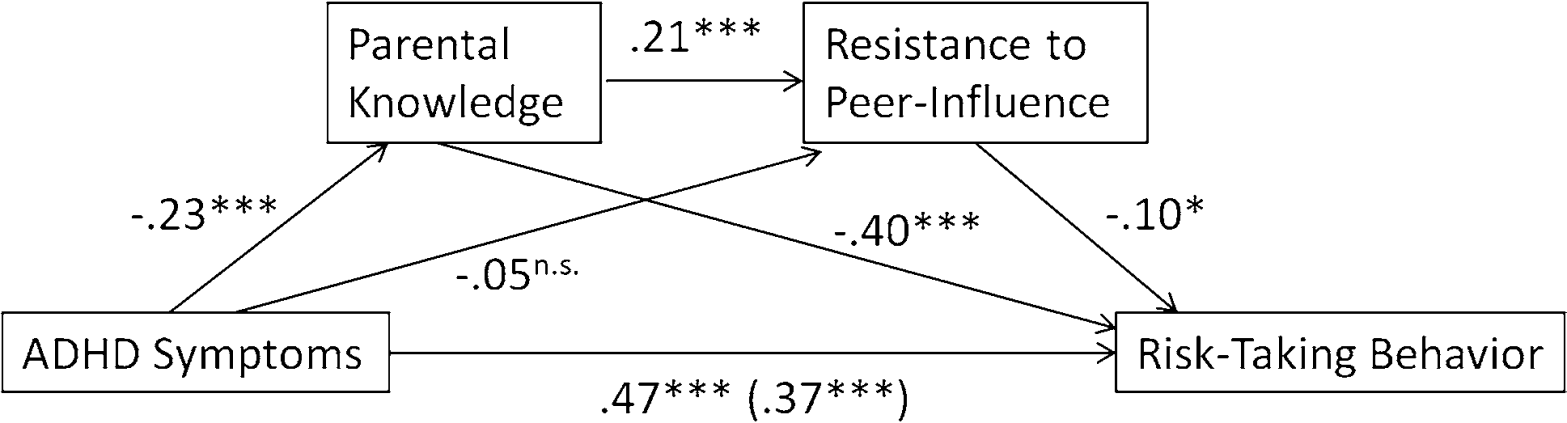
Mediation model: standardized regression coefficients for the relationship between ADHD Symptoms and Risk-Taking Behavior as mediated by Parental Knowledge and Resistance to Peer Influence. The direct effect of ADHD Symptoms on Risk-Taking Behavior, controlling for Parental Knowledge and Resistance to Peer Influence, is in parentheses; * *p* < .05, ** *p* < .01, *** *p* < .001

As pre-registered, we hypothesized that parental knowledge and resistance to peer influence are both mediating the association between ADHD symptoms and RTB. The serial mediation model is depicted in Figure 2. First, we expected that a higher number of ADHD symptoms predicted more engagement in RTB, lower parental knowledge and a lower resistance to peer influence. Second, we expected that higher parental knowledge predicted a larger resistance to peer influence, and we expected that both parental knowledge and resistance to peer influence predicted lower engagement in RTB. Third, we expected that both parental knowledge and resistance to peer influence mediated the link between ADHD symptoms and RTB.

### Methods

#### Preregistration

This study was preregistered on AsPredicted (#15669; https://aspredicted.org/bj73g.pdf). Note that the pre-registration also contains information about another study, conducted in the same sample, which will be reported in a separate article.

#### Participants

Participants were 313 late adolescents (*see* Table 1), recruited at their school. There were no exclusion criteria. Forty-nine participants (15.7%) indicated that they had been diagnosed with ADHD (any presentation) in the past, 19 of them (6.1%) were using stimulant medication at the time of testing. A total of 18.8% of the adolescents had a clinical ADHD score and reported significant impairment. A total of 62.6% of the adolescents indicated that they were never diagnosed with any disorder. Furthermore, lifetime diagnoses of anxiety disorders (9.3%), autism spectrum disorders (2.9%), disruptive behavioral disorders (0.3%), mood disorders (10.2%), eating disorders (2.9%), obsessive-compulsive disorders (1.6%), dyslexia (10.2%), substance use disorders (1.3%) and tic disorders (0.3%) were reported. The large majority of the participants were enrolled in secondary vocational education in the Netherlands, and therefore intelligence of all participants was likely to be average. Ethnic identity of the participants was predominantly Dutch (93% Dutch, 7% Surinamese, 3.8% Antillean, 3.8% Moroccan, 3.5% Turkish, 1.9% Kurdish, 17.9% other)^2^. The study was approved by the IRB of the University of Amsterdam. All participants gave written informed consent.

#### Materials

For ADHD symptomatology, RTB and parental knowledge, the same instruments were used as in study 1. In the current sample, internal consistency of the measures was good (ADHD Self-report Scale: *α* = .90; Parental Knowledge Scale: *α* = .87; RTB Questionnaire: *α* = .86).

##### Resistance to Peer Influence scale

The Resistance to Peer Influence scale (RPI [50]) is a self-report questionnaire that consists of 10 items. On each item, participants first indicated which of two statements described them better and then indicated whether this statement described them totally or partially. An example of an item is “Some children take more risks when they are with their friends than when they are alone – Other children do not take more risks when they are with their friends than when they are alone”. Answers were coded on a 4-point scale, ranging from completely agreeing with one statement to completely agreeing with the other statement. Scores potentially range from 10 to 40, with higher scores reflecting more resistance to peer influence. Internal consistency of the Dutch translation was acceptable [51]. In the current sample, the internal consistency was just below what would be considered satisfactory (*α* = .66) and therefore results should be interpreted cautiously. However, internal consistency was similar to two studies investigating the psychometric properties of the RPI, with alpha’s between .70 and .75 [50, 51].

#### Procedure

Participants were first briefed about the study in class. Those willing to participate gave written informed consent and were tested on their own laptop while being in their classroom. Two research assistants and the teacher were available for questions during the assessment. A small minority of the participants forgot their laptop and completed the questionnaires using paper-and-pencil. The duration of the session was approximately 30 minutes.

#### Data-analysis

As pre-registered, a mediation analysis was performed using the SPSS Process Macro, model 6 (model 6 [48], default 5000 samples bootstrapping, standardized values), with ADHD symptoms as independent variable, parental knowledge as first mediator, resistance to peer influence as second mediator and RTB as dependent variable. Outliers and missing data were handled as pre-registered. All variables were standardized before analysis.

### Results

Two participants did not complete the resistance to peer influence scale and were therefore not included in the analyses. Hence, the final sample size is 311.

#### Exploratory correlation analyses

Correlations between all variables were calculated. As ADHD symptoms, parental knowledge, RTB and resistance to peer influence were not distributed normally (Kolmogorov-Smirnov *p*’s < .01), Spearman’s correlation analyses were performed. All correlations were as expected: ADHD symptoms correlated positively with RTB and negatively with parental knowledge and resistance to peer influence; parental knowledge correlated negatively with RTB and positively with resistance to peer influence; resistance to peer influence correlated negatively with RTB (*see* Table 3).

**Table 3:** Spearman’s correlations between ADHD Symptoms, Parental Knowledge (PK), Resistance to Peer Influence (RPI) and Risk-Taking Behavior (RTB). All variables reflect sum scores. * *p* < .05, *** *p* < .001

	ADHD symptoms	PK	RPI	RTB
ADHD Symptoms	−			
Parental Knowledge	−.24***	−		
Resistance to Peer Influence	−.12*	.24***	−	
Risk-Taking Behavior	.46***	−.45***	−.21***	-

#### Pre-registered mediation analyses

Results are graphically depicted in Figure 2. ADHD symptoms significantly predicted parental knowledge, *b* = -.23, *t*(309) = -4.08, *p* < .001; adolescents with more ADHD symptoms reported less parental knowledge. In a model that contained ADHD symptoms and parental knowledge, ADHD symptoms did not significantly predict resistance to peer influence, *b* = -.05, *t*(308) = -.86, *p* = .39, whereas, parental knowledge did, *b* = .21, *t*(308) = 3.61, *p* < .001, indicating that more parental knowledge was related to a higher resistance to peer influence.

In a model that also contained ADHD symptoms and resistance to peer influence, parental knowledge significantly predicted RTB, *b* = -.40, *t*(307) = -8.17, *p* < .001. In the same model, resistance to peer influence also predicted RTB, *b* = -.10, *t*(307) = -2.14, *p* = .034. This indicates that less parental knowledge and lower resistance to peer influence were both related to enhanced RTB (see Fig. 2).

The total effect of ADHD symptoms predicting RTB was significant, *b* = .47, *t*(309) = 8.88, *p* < .001. Also after taking into account all indirect effects, ADHD symptoms still predicted RTB, *b* = .37, *t*(307) = 7.60, *p* < .001.

Three indirect effects were derived from the model. First, the indirect effect of ADHD symptoms on RTB through parental knowledge was significant, as the bootstrap derived 95% confidence interval did not contain zero (.037, .155). The indirect effect explained 19.4% of the total effect, as established by dividing the standardized *b* of the indirect effect by the standardized *b* of the total effect [49]. Second, the indirect effect of ADHD symptoms on RTB through resistance to peer influence was not significant (-.009, .025), and only explained 1.1% of the total effect. Third, the indirect serial effect of ADHD symptoms on RTB through parental knowledge and then resistance to peer influence was significant (.0002, .013), although it only explained 1.0% of the total effect. The overall model including ADHD symptoms, parental knowledge, resistance to peer influence and RTB was also significant, *F*(3,307) = 60.26, *p* < .001, *R*^*2*^ = .37.

As an additional control, all regression analyses were also performed using bootstrapping of 5000 samples, as the data distribution was not normal: all *b*- and *p*-values were highly similar.

#### Outliers

Based on median absolute deviation [52], as pre-registered, 28 outliers were detected (8 on RTB, 7 on ADHD symptoms, 2 on resistance to peer influence and 11 on parental knowledge). Some participants had an outlier score on multiple measures. Finally, 25 participants were excluded. The same analyses, after exclusion of outliers, yielded roughly the same results. The only pathway that changed in terms of significance was the effect from resistance to peer influence to RTB, which was no longer significant after excluding outliers.

#### Explorative analyses

As an additional check, we also analyzed the serial mediation model with resistance to peer influence as first mediator and parental knowledge as second mediator (i.e., the order of the mediators was reversed). In that case, all effects were similar, except that the serial indirect effect was no longer significant. Thus, parental knowledge predicting resistance to peer influence mediated the link between ADHD symptoms and RTB, but resistance to peer influence predicting parental knowledge did not mediate the link between ADHD symptoms and RTB.

We also analyzed both mediators in single, separate mediation analyses, to establish their effects without the influence of the other mediator. Results were highly similar to the first serial mediation analysis: the indirect effect of ADHD symptoms on RTB through parental knowledge was significant, the indirect effect through resistance to peer influence was not significant.

### Discussion

The second study replicated the findings of the first study: parental knowledge partially mediated the association between ADHD symptoms and RTB. The pre-registered design of this replication increases the confidence in this finding [25]. Furthermore, the effect of parental knowledge on the association between ADHD symptoms and RTB seems stronger than the effect of resistance to peer influence. Participants with more ADHD symptoms did not indicate to be more susceptible to peer influence, and resistance to peer influence had a smaller effect on RTB than parental knowledge, although the low internal consistency and thus low reliability of the RPI scale could also explain these findings. However, resistance to peer influence had a more circuitous effect: parental knowledge and resistance to peer influence were associated, and together mediated the link between ADHD symptoms and RTB.

## Study 3

The first two studies demonstrated the importance of parental knowledge in understanding the link between ADHD symptoms and RTB. In the third study, we tested whether the influence of parental knowledge extended to other ADHD-related problems in adolescence that cause impairment, like homework problems. More specifically, we expected that (I) ADHD symptoms correlated positively with homework problems; (II) ADHD symptoms correlated negatively with parental knowledge; (III) parental knowledge correlated negatively with homework problems and (IV) there was an indirect effect of ADHD symptoms on homework problems through parental knowledge.

### Methods

#### Preregistration

The pre-registration was identical to Study 2 (https://aspredicted.org/bj73g.pdf).

#### Participants

Participants were 315 late adolescents (*see* Table 1), recruited at their school. There were no exclusion criteria. 56 participants (17.8%) indicated that they had been diagnosed with ADHD (any presentation) in the past, 18 of them (5.7%) were using stimulant medication at the time of testing. A total of 19.4% of the adolescents had a clinical ADHD score and reported significant impairment. A total of 60.6% of the adolescents indicated that they were never diagnosed with any disorder. Furthermore, lifetime diagnoses of anxiety disorders (7.3%), autism spectrum disorders (3.5%), disruptive behavioral disorders (0.3%), mood disorders (12.1%), eating disorders (2.9%), obsessive-compulsive disorders (1.9%), dyslexia (10.2%) and substance use disorders (0.6%) were reported. The large majority of the participants were enrolled in secondary vocational education in the Netherlands, and therefore intelligence of all participants was likely to be average. Ethnic identity of the participants was predominantly Dutch (92.1% Dutch, 7.6% Surinamese, 4.8% Antillean, 5.4% Moroccan, 6.0% Turkish, 3.5% Kurdish, 20.3% other)^2^. The study was approved by the IRB of the University of Amsterdam. All participants gave written informed consent. Participants of Study 2 did not perform in Study 3 and vice versa.

#### Materials

For ADHD symptomatology and parental knowledge, the same instruments were used as in study 1 and 2. In the current study, internal consistency of both measures was excellent (ADHD Self-report Scale: *α* = .92; Parental Knowledge Scale: *α* = .89).

##### Homework Problems Checklist

The Dutch translation of the Homework Problems Checklist [53] was used to assess homework problems. As the original questionnaire is parent-reported, for the current study, the items were rewritten to make it suitable for self-report. An example of a question is “I am easily frustrated by homework assignments”. The questionnaire consisted of 20 items answered on a 4-point Likert scale. Scores potentially range from 0 to 60, with higher scores reflecting more homework problems. The original parent-reported version has good psychometric properties [53, 54]. In this study, internal consistency was excellent, *α* = .92.

#### Procedure

The procedure was identical to study 2.

#### Data-analysis

As pre-registered, a mediation analysis was performed using the SPSS Process Macro (model 4 [48], default 5000 samples bootstrapping, standardized values), with ADHD symptoms as independent variable, parental knowledge as mediator and homework problems as dependent variable. Outliers and missing data were handled as was pre-registered.

### Results

#### Exploratory correlation analyses

Correlations between all variables were calculated. As ADHD symptoms, parental knowledge and homework problems were not distributed normally (Kolmogorov-Smirnov *p*’s < .01), Spearman’s correlation analyses were performed. As expected, ADHD symptoms correlated positively with RTB and negatively with parental knowledge; parental knowledge correlated negatively with homework problems (*see* Table 4).

**Table 4:** Spearman’s correlations between ADHD Symptoms, Parental Knowledge and Homework Problems. All variables reflect sum scores. *** *p* < .001

	ADHD symptoms	Parental knowledge	Homework problems
ADHD symptoms	−		
Parental knowledge	−.31***	−	
Homework problems	.79***	−.36***	−

#### Pre-registered mediation analyses

**Fig. 3:**
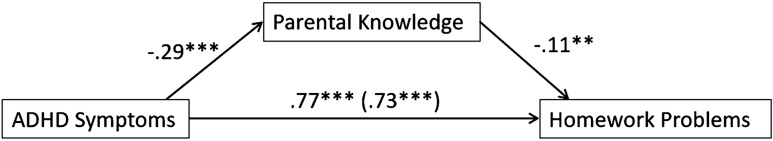
Mediation model: standardized regression coefficients for the relationship between ADHD Symptoms and Homework Problems as mediated by Parental Knowledge. The direct effect of ADHD Symptoms on Homework Problems, controlling for Parental Knowledge, is in parentheses; * *p* < .05, ** *p* < .01, *** *p* < .001

ADHD symptoms significantly predicted parental knowledge, *b* = -.29, *t*(313) = -5.36, *p* < .001; adolescents with more ADHD symptoms reported less parental knowledge. In a model that included ADHD symptoms, parental knowledge significantly predicted homework problems, *b* = -.11, *t*(312) = -3.16, *p* = .002; adolescents reporting more parental knowledge experienced less homework problems (see Fig. 3).

The total effect of ADHD symptoms predicting homework problems was significant, *b* = .77, *t*(313) = 22.97, *p* < .001. Also after taking into account the mediating role of parental knowledge, ADHD symptoms still predicted homework problems, *b* = .73, *t*(312) = 21.38, *p* < .001. The indirect effect was significant, as the bootstrap-derived confidence interval did not contain zero (.009, .061) and the overall model including ADHD symptoms, parental knowledge and homework problems was significant, *F*(2,312) = 276.32, *p* < .001, *R*^2^ = .64. The indirect effect explained 4.1% of the total effect, as established by dividing the standardized *b* of the indirect effect by the standardized *b* of the total effect [49].

Again, as an additional check, all regression analyses were also performed using bootstrapping of 5000 samples, as the data distribution was not normal for some variables: all *b*- and *p*-values were highly similar.

#### Outliers

Based on median absolute deviation [52], as pre-registered, 44 outliers were detected (11 on ADHD, 17 on parental knowledge and 16 on homework problems). Some participants had an outlying score on multiple measures. Finally, 36 participants were excluded. After excluding these outliers, results were almost similar and no effects changed in terms of significance.

## Discussion

The third study extended previous findings in the domain of risk-taking behavior by demonstrating that parental knowledge also mediated the link between ADHD symptoms and homework problems.

### Explorative analyses study 1-3

As explorative analyses, all mediation analyses reported above were also performed for symptoms of inattention and symptoms of hyperactivity/impulsivity separately. Effects reported above were highly similar for both symptom clusters of ADHD in terms of statistical significance as well as magnitude. Visual representations of these explorative mediation models can be found in Supplementary Materials 2.

## General discussion

In three consecutive studies, we demonstrated that parental knowledge (i.e., “knowing where, how and with whom children spend their time” [13]) mediated the link between ADHD symptoms and different domains of impairment. In the first study, we replicated earlier findings on the mediating influence of parental knowledge on the link between ADHD symptoms and risk-taking behavior (RTB) [16]. Similar to the original study, parental knowledge mediated the association between ADHD symptoms and RTB. The current study, performed by an independent research group, in a different country, using a slightly different age range and using a 2.5 times larger sample size than the original study (cf. guidelines [41]), increases the robustness and strengthens the generalizability of the original findings.

In the second preregistered study, to compare different social influences on RTB, self-reported resistance to peer influence was additionally investigated. Parental knowledge was associated with resistance to peer influence, which together mediated the link between ADHD symptoms and RTB. That is, low parental knowledge, which was associated with ADHD, was not only directly associated with increased RTB, it was also associated with a lower resistance to peer influence, which in turn predicted RTB as well. This highlights the importance of investigating the joint influence of different social aspects on RTB, and suggests that those adolescents with low parental knowledge are particularly susceptible to peer influence.

In the third study, we investigated whether the importance of parental knowledge was specific for the association between ADHD symptoms and RTB or also generalized to homework problems. Crucially, parental knowledge mediated the link between ADHD symptoms and homework problems, which suggests that the influence of parental knowledge extends to other domains than RTB. However, the indirect effect of parental knowledge in this study was substantially smaller than in the first two studies.

### Clinical implications

The robust mediating role of parental knowledge in the association between ADHD symptoms and behavior that often causes impairment in ADHD like RTB and homework problems suggests that increasing parental knowledge may be useful for treatment of adolescents high on ADHD symptoms and RTB, and for the prevention of RTB in this group. Parental knowledge can originate from three different sources: solicitation by parents (i.e., asking/requesting information about adolescents’ activities/behavior), control from parents (i.e., setting limits, restricting activities), and adolescent disclosure (i.e., voluntarily sharing information with parents) [13]. From these three, a lack of disclosure by the adolescent was a strong predictor of delinquency and norm-breaking behavior later in time, whereas parental solicitation and control were not [55], indicating the importance of voluntary disclosure by the adolescent in parent-child relationships. Potentially, adolescents’ disclosure may improve the emotional climate within a family, making it more likely that youths disengage from RTB [55]. But at the same time the emotional climate has a great influence on adolescents’ disclosure. Voluntary disclosure is most likely to emerge in a “warm and responsive parent-child relationship” [56] and enhancing these aspects of the parent-child relationship may therefore be instrumental. In sum, improvement of the parent-child relationship is likely to increase child disclosure, resulting in increased parental knowledge, the latter being predictive of lower levels of RTB [55]. However, it should be noted that these three sources of parental knowledge were not measured directly in the current set of studies. Therefore, we encourage future studies to assess what sources of parental knowledge are associated to the link between ADHD and RTB/homework problems, and to test the hypothesis based on previous studies that disclosure should be the main focus of intervention.

Similarly, parental knowledge may also be an important target in the treatment of homework problems in adolescents high on ADHD symptoms. For example, a promising new treatment module for adolescents with ADHD called STAND included parents in all sessions with the adolescents, and was more effective than treatment as usual as measured on core symptoms as well as academic impairment [33]. Some of the presumed underlying mechanisms explaining the success of this treatment were an increase in monitoring skills from parents and reductions in conflicts between adolescents and parents [33]. Similarly, another study demonstrated that homework problems in typically developing adolescents decreased after an intervention aimed at parental monitoring, stressing the importance of parental involvement in homework interventions [35].

### Strengths and limitations

The first strength of the current investigation is the methodological rigor. The mediating role of parental knowledge in the link between ADHD symptoms and ADHD-related problems was established in three consecutive studies. Although the focus of research is more often on discovering novelty, replication is crucial for the generalizability of the findings and for the progress of science, as this provides greater confidence about findings [57, 58]. Furthermore, the design and methodology of study 2 and study 3 were pre-registered. Pre-registration prevents researchers from flexible data-analytic strategies, which also results in a larger confidence in the findings [25].

A second strength is that we systematically added assessments of additional variables to the initial investigation of the association between ADHD symptoms, parental knowledge and risk-taking behavior. By doing this we demonstrated that (I) parental knowledge was a stronger contributor in the association between ADHD symptoms and RTB than resistance to peer influence, (II) parental knowledge is associated with resistance to peer influence, together mediating the link between ADHD symptoms and RTB and (III) parental knowledge also mediated the association between ADHD symptoms and homework problems. From these three consecutive studies, we conclude that parental knowledge is a crucial mechanism in understanding ADHD-related impairment across domains.

A limitation of the design of all studies is that data is cross-sectional. The correlational nature of the data prevents drawing causal conclusions. It is therefore possible that the direction of some of the effects may be different from how it was analyzed: For example, low parental knowledge could be a consequence of RTB rather than an antecedent, as it is likely that adolescents engaging in RTB do not disclose about these behaviors to their parents. However, the direction of the variables in all of our models was based on a wealth of literature. Several meta-analytic and prospective cohort studies demonstrated that ADHD status predicts both RTB (e.g., [59], [60]) and academic problems [61, 62]. Also, the causal link between childhood ADHD and later parenting problems is supported by many studies (e.g., [63]), and longitudinal studies demonstrated that parental monitoring negatively predicts later RTB like delinquency and substance abuse [55, 64, 65], although some studies observed effects in opposite directions (e.g., [66]). Future longitudinal studies are needed to further establish the causal nature of the association between ADHD symptoms, parental knowledge and different domains of ADHD-related problems like RTB and homework problems. Similarly, the observed mediating effects of parental knowledge could potentially be ascribed to the correlation between parental knowledge and other factors that may be related to the link between ADHD and RTB, such as socio-economic status, parental intelligence and openness of the family climate [67]. Intervention studies targeting parental knowledge in adolescents high on ADHD symptoms could elucidate whether RTB declines as a consequence, or whether other variables are responsible for the mediating effects of parental knowledge as established in the current study.

The current study relied on self-report only which might have caused biased (e.g., halo effects) or socially desirable answers. However, for the central construct of this study—parental knowledge—similar response patterns were observed for self- and other report [13], and previous work demonstrating similar results to the current study also relied on self-report [16]. The Resistance to Peer Influence scale (RPI) was specifically developed as a self-report scale with minimal influence of socially desirable responding [50]. For ADHD symptomatology and homework problems, reliance on self-report might be more problematic: For example, parents have been shown to be more valid reporters of ADHD symptoms than adolescents [68]. Although the strong correlation between ADHD symptoms on the one hand and RTB and homework problems on the other hand may be interpreted as an indication of convergent validity, future studies should ideally incorporate parental measures of ADHD and homework problems alongside self-report measures.

On a related note, internal consistency of the RPI was low, although similar to other RPI studies [50], [51]. Therefore, interpretation of the results related to resistance to peer influence warrants caution. A previous study on the structure of the RPI indicated that the scale not only measures resistance to peer influence but also the tendency to respond extremely [69]. This intermixing of two factors may have reduced internal consistency. Future studies may therefore adopt the Dekkers et al. [69] modeling approach to disentangle these two separate components. As an alternative, experimental paradigms (e.g., [19], [70]) for assessing susceptibility to peer influence could also be considered in future research.

Finally, parental knowledge only partially mediated the link between ADHD symptoms and RTB/homework problems. The effect ratio (indirect effect / total effect) indicated that the indirect effect of parental knowledge explained 10.5% and 19.4% of the link between ADHD symptoms and RTB in the first two studies, respectively. This is smaller than the 34.3% that was found by Pollak and colleagues [16], but is still substantial, especially given the broad range of factors that have been associated with the link between ADHD and RTB [7]. As the link between ADHD symptoms and RTB is well-established in the literature [5, 7, 71], we argue that effects of this magnitude are meaningful.

However, apart from these indirect effects, ADHD symptomatology alone has a major contribution in the development of both RTB and homework problems, and treating core symptoms of ADHD should always be an aim in treatment. Nevertheless, the current set of studies and the highly consistent patterns of outcomes highlight the importance of parental knowledge in the interplay between ADHD symptoms and some of the problems related to these symptoms.

To sum up, three consecutive studies with large and independent samples demonstrated the importance of parental knowledge in the association between ADHD symptomatology and related domains of impairment. Targeting parental knowledge in treatment of adolescents with ADHD may be a promising way to go.

## Electronic supplementary material

Below is the link to the electronic supplementary material.

Supplementary materials (DOCX 210 kb)

## Footnotes

1 Note that by using the term mediation, we certainly not mean to make any causal inferences. We are aware of the debate in statistical literature on this topic [48, 72]. Where we mention mediator, this can be interpreted as an indirect effect.

2 Participants could indicate to what socio-cultural group they feel to belong. As multiple answers were possible for each participant, the sum exceeds 100%.
