# Supplementary material for: The importance of parental knowledge in the association between ADHD symptomatology and related domains of impairment
Source: Eur Child Adolesc Psychiatry. 2020 Jul 22;30(4):657–69. doi: 10.1007/s00787-020-01579-4 (PMC8041705; doi:10.1007/s00787-020-01579-4)
Supplement: Supplementary file 1 — Supplementary materials (DOCX 210 kb) [file 787_2020_1579_MOESM1_ESM.docx]

**Supplementary Materials 1: Risk Taking Questionnaire**

| **How often do you engage in the following behavior?** |  |
| --- | --- |
| 1. Fighting | Never 0 1 2 3 4 Every week |
| 1. Smoking cigarettes | Never 0 1 2 3 4 Every week |
| 1. Using soft drugs (weed, hash etc.) | Never 0 1 2 3 4 Every week |
| 1. Using hard drugs (xtc, cocaine, etc.) | Never 0 1 2 3 4 Every week |
| 1. Using smartphone while cycling/driving scooter | Never 0 1 2 3 4 Every week |
| 1. Cycling or scooter driving under influence of alcohol/drugs | Never 0 1 2 3 4 Every week |
| 1. Having unprotected sex | Never 0 1 2 3 4 Every week |
| 1. Stealing | Never 0 1 2 3 4 Every week |
| 1. Gambling online or in real life (e.g. online betting, casino) | Never 0 1 2 3 4 Every week |
| 1. Disobeying teachers at school | Never 0 1 2 3 4 Every week |
| 1. Disobeying parents | Never 0 1 2 3 4 Every week |
| 1. Eating dysfunctional (vomiting, fasting or gorging) | Never 0 1 2 3 4 Every week |
| 1. Breaking the law | Never 0 1 2 3 4 Every week |
| 1. Running away from home | Never 0 1 2 3 4 Every week |
| 1. Injuring self | Never 0 1 2 3 4 Every week |
| 1. Meeting someone from the Internet | Never 0 1 2 3 4 Every week |
| 1. Drive a car or a scooter without license | Never 0 1 2 3 4 Every week |
| 1. Sunbathing without sunscreen | Never 0 1 2 3 4 Every week |
| 1. Extreme sports (e.g. free running) | Never 0 1 2 3 4 Every week |
| 1. Binge drinking | Never 0 1 2 3 4 Every week |
| 1. Arriving home later than permitted | Never 0 1 2 3 4 Every week |
| 1. Driving or being on the back of a scooter without helmet | Never 0 1 2 3 4 Every week |
| 1. Copying answers from others during an exam | Never 0 1 2 3 4 Every week |
| 1. Ignoring a red light while walking/cycling/driving | Never 0 1 2 3 4 Every week |
| 1. Lying against friends/parents | Never 0 1 2 3 4 Every week |
| 1. Copying homework/plagiarism | Never 0 1 2 3 4 Every week |
| 1. Sharing a friends’ secret | Never 0 1 2 3 4 Every week |
| 1. Driving in a car without seatbelt | Never 0 1 2 3 4 Every week |

**Supplementary Materials 2: Explorative mediation analyses**

**Study 1**

**
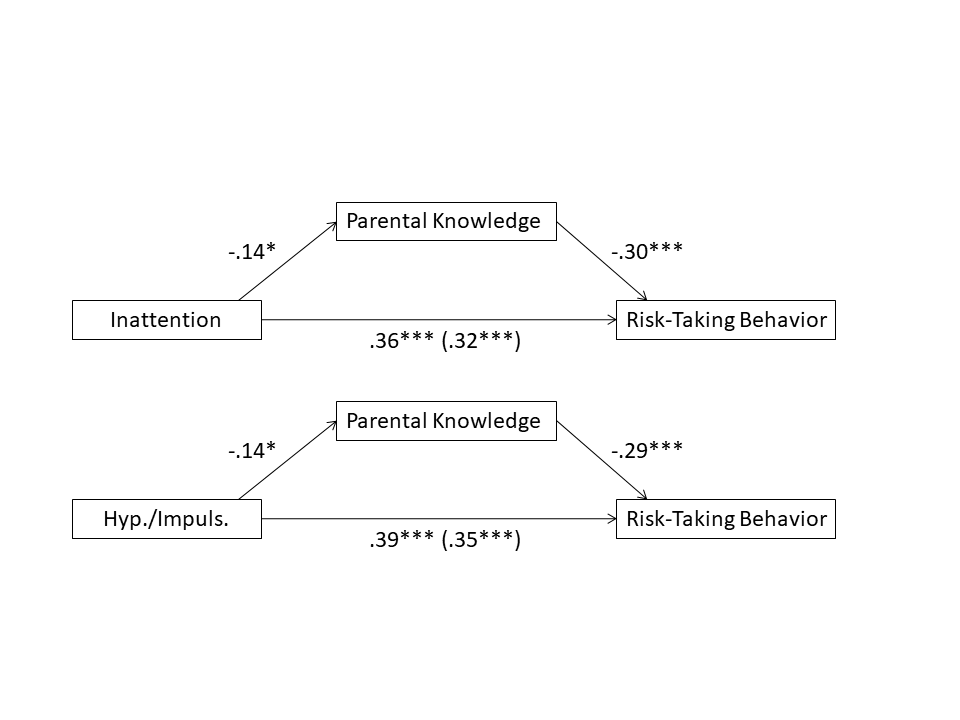
**

**Figure S1.** Mediation models as calculated in Study 1 for inattention (upper panel) and hyperactivity/impulsivity (lower panel) symptoms separately. The indirect effects of inattention and hyperactivity/impulsivity on risk-taking behavior through parental knowledge were both significant.

**Study 2**

**
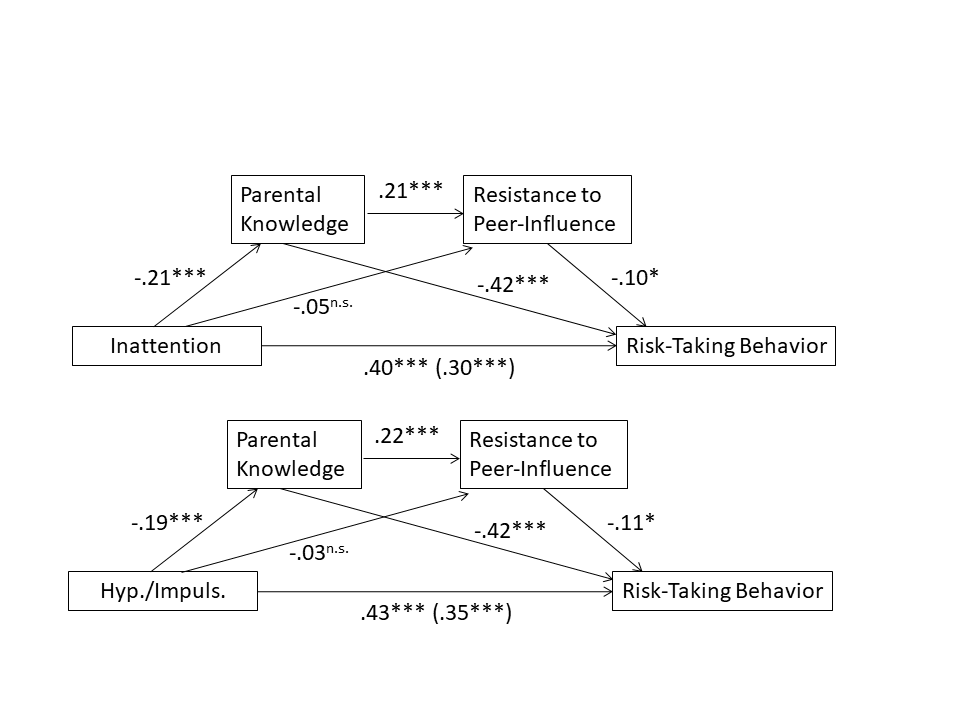
**

**Figure S2.** Mediation models as calculated in Study 2 for inattention (upper panel) and hyperactivity/impulsivity (lower panel) symptoms separately. Indirect effects of inattention and of hyperactivity/impulsivity on risk-taking behavior through parental knowledge were significant, indirect effects through resistance to peer influence were not significant; indirect serial effects were significant in both models.

**Study 3**

**
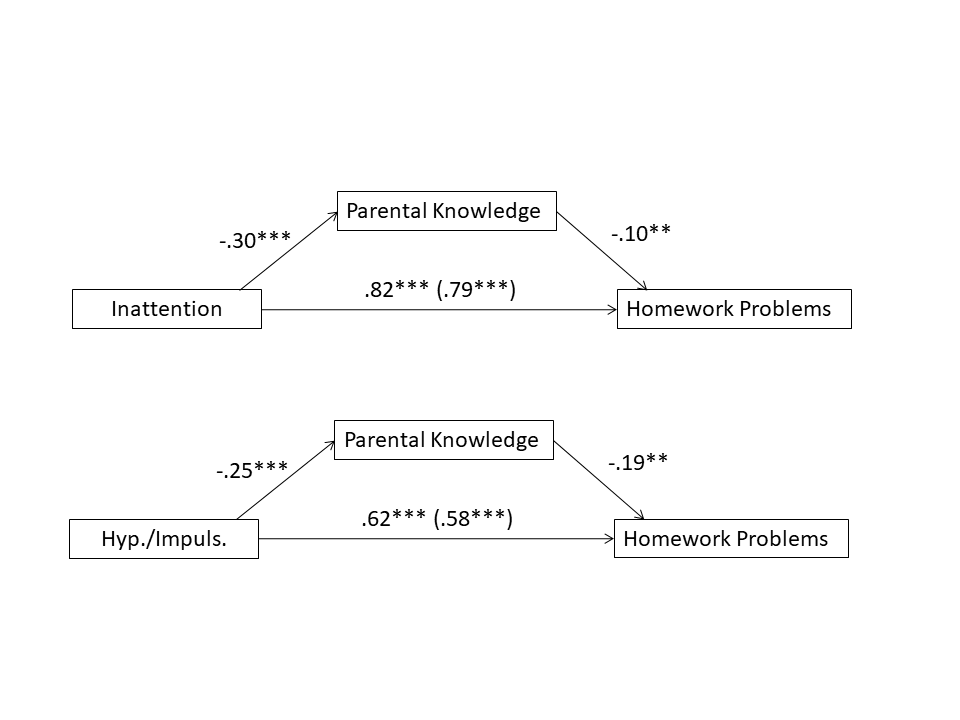
**

**Figure S3.** Mediation models as calculated in Study 3 for inattention (upper panel) and hyperactivity/impulsivity (lower panel) symptoms separately. The indirect effects of inattention and hyperactivity/impulsivity on homework problems through parental knowledge were both significant.
